# 

**Published:** 1821-06-01

**Authors:** 


### THE
### Medico-Chirurgical Review,
### AND
### JOURNAL
### OF
### MEDICAL SCIENCE.
### (Quarterly)

EXHIBITING A COMPREHENSIVE ANALYTICAL RECORD OF PROGRESSIVE MEDICINE AND SURGERY;

*EQUALLY ADAPTED TO ALL RANKS OF THE PROFESSION.*

CONDUCTED BY

ASSOCIATED PHYSICIANS AND SURGEONS;

AND SUPERINTENDED BY

JAMES JOHNSON, M. D.

MEMBER OF THE ROYAL COLLEGE OF PHYSICIANS OF LONDON.

(Analytical Series.)

S. G. O. LIBRARY 20568

VOLUME II. for 1821-2.

Nec tibi quid liceat sed quid fecisse decebit

Occurrat mentemque domat respectus honesti. CLAUD.

London:
Printed by G. HAYDEN, Little College Street, Westminster,
FOR THE EDITOR,
No. 58, Spring Gardens,
[Where Books for Review and Critical Communications, &c. *(post paid)* are to be directed.]

Published by BURGESS & HILL, Windmill Street, Haymarket; BALDWIN, CRADDOCK, & JOY, Pater-Noster Row; BLACK, PARBURY, & Co. Leadenhall Street; HIGHLEY, and T. and G. UNDERWOOD, Fleet Street; J. CALLOW, Princes Street, Soho; COX & SON, Borough; ANDERSON, West Smithfield; JOHN COX, Berners Street, Oxford Street; ADAM BLACK, Edinburgh; HODGES and M'ARTHUR, Dublin; Messrs. TREUTTLE & WURTZ, Paris and Strasburgh.

1822.

Bibliographical Record - - - - - - - - - - - - - 230

Notices, &c. &c. &c.- - - - - - - - - - - - - - 235

Additional Subscribers - - - - - - - - - - - - - 236

---

On the 1st July will be published, Third Edition, greatly enlarged and improved, "The Influence of Tropical Climates on European Constitutions." By James Johnson, M. D.

### BIBLIOGRAPHICAL RECORD.

### *Books Received for Review since March 1st. 1821.*

1. A Dissertation on the Treatment of Morbid Local Affections of Nerves: to which the Jacksonian Prize was adjudged by the Royal College of Surgeons. By JOSEPH SWAN, Member of the Royal College of Surgeons, and Surgeon to the Lincoln County Hospital. One vol. 8vo, pp. 196, with a coloured plate of the nerves of the face. London, 1820.

☞ *See page 63.*

---

2. An Account of a new Method of making dried Anatomical Preparations. By JOSEPH SWAN, Member of the Royal College of Surgeons, and Surgeon to the Lincoln County Hospital. Second Edition, enlarged. One vol. 8vo, pp. 132. London, 1820.

---

3. A Treatise on the Art of Cupping: in which the History of that Operation is traced: the Complaints in which it is useful indicated, and the most approved method of performing it described. By THOMAS MAPLESON, Cupper to His Majesty. *Second Edition,* considerably improved. One vol. duodecimo, pp. 96. Callow, 1821.

☞ *The author of this useful little work has long been known as one of the most expert cuppers in this metropolis—an expertness which we have often personally witnessed. From the now increasing attention to* local *blood-letting we can recommend this little volume to our brethren, though that is hardly necessary, since its merits are already appreciated by the public, as evinced by a new edition.*

---

4. A Monthly Journal of Popular Medicine, explaining the Nature, Causes, and Prevention of Disease, the immediate Management of Accidents, and the Means of preserving Health. Conducted by CHARLES THOMAS HADEN, Surgeon to the Chelsea and Bromton Dispensary, &c. No. I, for March, No. 2, for April, and No. 3, for May, 1821. Octavo, 48 pages each number.

---

5. Practical Observations on the Use of Oxigen or Vital Air, in the Cure of Diseases: to which are added, a few Experiments on the Vegetation of Plants, illustrated with five engravings. By DANIEL HILL, M. D. Surgeon, Honorary Member of the Medical Society at Guy's Hospital, and Fellow of the Horticultural Society. *Second Edition, with an Appendix.* One vol. 8vo, pp. 102. London, 1821.

6. Discours sur le préeminence du Systême nerveux dans l'economie animale, et l'importance d'une étude approfondie de ce systême; prononcé par JEAN FREDIRIC LOBSTEIN, Professeur D'Anatomic Pathologique, et Directeur du Museum de Strasbourg. Strasbourg, 1821. *De la part de M. Breschet.*

---

7. Bulletins de la Faculté de Medecine, de Paris. Paris, No. X. 1820. *De la part de M. Breschet.*

---

8. REVUE MEDICALE, HISTORIQUE et PHILOSOPHIQUE. Tome Quatrieme—February 1821, containing Moreau de Jonnes on the Yellow Fever—Duval on the Teeth—Montegre on Hæmorrhoids —Lallemand and Rostan on Softening of the Brain—St. Laurens on Gastric Erysipelas—Bulletins of the Medical Society of Emulation, &c. &c.

---

9. A Letter addressed to the Legislature on Vaccination. By J. TAYLOR, M. D. Physician Extraordinary to his Royal Highness the Duke of Clarence. Octavo, sewed, pp. 16. London, 1821.

☞ *A zealous appeal to Parliament in favour of an investigation into the late supposed failures of vaccination, in order that public confidence may be restored, and the spread of the variolous pest restrained.*

---

10. A descriptive Catalogue of the British Specimens deposited in the Geological Collection of the Royal Institution. One vol. 8vo, pp. 212. By Professor W. T. Brande.

☞ *The collection of which this valuable volume exhibits a descriptive catalogue, was commenced several years ago by the present distinguished president of the Royal Society, and has since been increased and rendered much more complete by the able and indefatigable gentleman who now fills Sir H. Davy's former chair, at the Royal Institution.*

---

11. The History of the Small Pox. By JAMES MOORE, Member of the Royal College of Surgeons, London, Director of the National Vaccine Establishment, &c. &c. One vol. 8vo, pp. 312, with a plate.

☞ *This is the most valuable and erudite history of small pox, its treatment, and counteraction, which we possess.*

---

12. Reports of the pestilential Disorder of Andalusia, which appeared at Cadiz, in the years 1800, 1804, 1810, and 1813; with a detailed Account of the fatal Epidemic, as it prevailed at Gibraltar, during the autumnal months of 1804; also, Observations on the remitting and intermitting Fever, made in the Mililary Hospitals at Colchester, after the return of the troops from the expedition to Zealand in 1809. By Sir JAMES FELLOWES, M. D. Fellow of Caius and Gonville College, Cambridge; Fellow of the Royal College of Physicians, London, &c. &c. &c. One vol. 8vo, pp. 484.

☞ *This is one of those interesting volumes which have resulted from the experience of our medical officers during the late war. We have given a full review of it in a former series of this Journal,*

---

13. A comprehensive Treatise upon the Symptoms, Consequences, Nature, and Treatment of Venereal or Syphilitic Diseases. Translated from the seventh French edition of F. SWEDIAUR, M. D. Two volumes in one, pp. 860. London, 1819.

☞ *This is by far the most complete treatise on the venereal disease in the English language, and ought to form a class book for the young practitioner, on entering on the duties of his professson. In a future number we hope to take more particular notice of the work.*

---

14. Observations on Sulphureous Fumigations, as a powerful Remedy in Rheumatism and Diseases of the Skin. By WILLIAM WALLACE, M. R. I. A. Member of the Royal College of Surgeons in Ireland; one of the Surgeons to the Charitable Infirmary; Surgeon to the Dublin Infirmary for curing Diseases of the Skin; Lecturer on Anatomy and Surgery, &c. One vol. 8vo, pp. 92. Dublin and London, 1821.

---

15. An Enquiry into the Nature and Treatment of Gravel, Calculus, and other Diseases connected with a deranged Operation of the Urinary Organs. By WILLIAM PROUT, M. D. F. R. S. Octavo, pp. 227. London, 1821.

☞ *See page* 89.

---

16. Practical observations on Fever, Dysentery, and Liver Complaints, as they occur amongst the European Troops in India: with Introductory Remarks, &c. &c. &c. By GEORGE BALLINGALL, M. D. F. R. S. E. Fellow of the Royal College of Surgeons, Edinburgh, and formerly Surgeon of H. M. 33d Regiment. Octavo, pp. 250.

---

17. A Manual of the Diseases of the Human Eye, intended for surgeons commencing practice; from the best national and foreign works, in particular those of Professor Beer: with the Observations of the Editor, Dr. Charles H. Weller. Berlin, 1819. Translated from the original German Work, and illustrated with Cases and Observations. By GEORGE C. MONTEATH, M. D. Member of the Royal College of Surgeons of London, Member of the Faculty of Physicians and Surgeons of Glasgow, and one of the Senior Surgeons to the Glasgow Royal Infirmary, &c. &c. Two volumes, 8vo, pp. 600, with five plates, and 37 beautifully coloured figures—nine representations of instruments. Glasgow, 1821.

☞ *The four following American Works have been received through the hands of our esteemed transatlantic friend and correspondent—Dr. Birckhead of Baltimore, viz.*

18. An Historical and Physical Sketch of a Malignant Epidemic, prevalent in Maryland, and some other States, within the last few years, &c. &c. By T. K. Wright, of Baltimore, 8vo, pp. 168.

---

19. Transactions of the Physico-Medical Society of New York, Vol. I. 8vo, pp. 446.

☞ *The contents of this interesting volume shall be made extensively known to the Profession, through the medium of this Review, as soon as possible.*

---

20. A Memoir on Contagion, more especially as it respects the Yellow Fever, read before the Medical and Chirurgical Faculty of Maryland. By Nathaniel Potter, M. D. &c. &c. Octavo, pp. 117.

---

21. A Treatise on Febrile Diseases, &c. By A. P. W. Philip, M. D. &c. &c. With *Notes and Additions*, by Nathan Smith, M. D. Professor of Physic, Surgery, and Obstetrics, in Yale College. Two volumes, 8vo.

---

22. An Inquiry into the Nature and Treatment of Gravel, Calculus, and other Diseases connected with a deranged Operation of the Urinary Organs. By William Prout, M. D. F. R. S. 8vo, pp. 227. Baldwin and Co. London, 1821.

☞ *See page* 89 *of the present number.*

---

23. Practical Observations on those Disorders of the Liver, and other Organs of Digestion, which produce the several forms and varieties of the Bilious Complaint. By Joseph Ayre, M. D. Physician to the General Infirmary, and to the Lying-in Charity, at Hull; and Senior Physician to the Hull and Sculcoates Dispensary. *Second Edition*, altered and enlarged, of the Essay on Marasmus. Octavo, pp. 270. Baldwin, London, 1821.

☞ *In the first volume of our Quarterly Series, p.* 399, *et seq. we gave a very full and comprehensive analysis of Dr. Ayre's valuable Treatise, which we strongly recommended to the Profession, predicting "that the work would be widely circulated and favourably received by the medical world." Our prediction has been verified, and we have only to say that this new edition is considerably improved, and the matter more concentrated than in the first edition.*

---

24. A Practical Treatise on the Inflammatory, Organic, and Sympathetic Diseases of the Heart; and also on Malformations of the Heart, Aneurism of the Aorta, Pulsation in Epigastrio, &c. &c.

By HENRY REEDER, M. D. Member of the Medical and Chirurgical Society of London, and Extraordinary Member of the Royal Medical Society of Edinburgh. One vol. octavo; pp. 276. London, 1821.

---

25. A Treatise on Indigestion and its Consequences, called Nervous and Bilious Complaints; with Observations on the Organic Diseases in which they sometimes terminate. By A. P. W. PHILIP, M. D. F. R. S. Ed. &c. One vol. 8vo, pp. 364. Underwood, 1821.

---

26. A Sketch of the History and Cure of Febrile Diseases; more particularly as they appear in the West Indies, among the Soldiers of the British Army. By ROBERT JACKSON, M. D. *Second Edition, with many additions.* Two vol. 8vo, pp. 880. London, 1820.

☞ *The first edition was published in* 1817. *The present is published*—" *because the author is desirous, before he may go hence, to leave the sum of what he has observed on the subject in a form that is intelligible and as easy to be understood as he is able to make it.*" Pref. *The matter indeed of these two volumes offers a most astonishing fund of information on the subject of fever, and no tropical visiter in particular, should proceed to his destination without possessing the work. The European physician too, will find that the veteran Jackson has anticipated almost every modern writer on fever, on all those points of pathology and practice in which we excel our forefathers. We hope soon to present our readers with an analytical view of the present work.*

---

27. Practical Observations on the Use of the Cubebs, or Java Pepper, in the Cure of Gonorrhœa, with Cases. By HENRY JEFFREYS, Esq. Senior Surgeon of the St. George's and St. James's General Dispensary; Assistant Surgeon to the Lock Hospital; and formerly a Surgeon in the Third Regiment of Guards. One vol. 8vo, pp. 68. London, 1821.

---

28. Reports of the Carlisle Dispensary, for the Year 1817, 1818, 1819, 1820. Medical Officers, Dr. John Heysham, Dr. Thomas Barnes, Dr. Thomas Blaniere, Mr. James Anderson.

☞ *We have been much pleased with these Reports, which do honour to the heads and hearts of the Medical Committee.*

---

29. Observations on the Varioloid Disease, or on Small Pox, under the form which it presents in persons previously vaccinated; illustrated by Cases and Experiments, published with a view to a true estimate of the value of vaccination. By WILLIAM STOKER, M. D. Licentiate of the College, and Member of the Association of the King's and Queen's College of Physicians in Ireland; Physician to the Fever Hospital, &c. &c. &c. Octavo, sewed, pp. 68. Dublin, 1821.

---

30. An Address to the Public on Vaccination, &c. By B. GRANGER, Surgeon. Octavo, sewed, pp. 34. Burton-on-Trent, 1821.

### ANNUNCIATIONS.

Mr. Lloyd, Senior Surgeon to the General Dispensary, Aldersgate Street, has in the press a Treatise on Scrofula, to which the Jacksonian Prize was adjudged.

The tenth number of the Quarterly Journal of Foreign Medicine and Surgery will be published on the 1st of June, 1821.

A third edition of Mr. Stowe's Toxicological Chart is just published.

*College Lectures, Warwick Lane.*

On the 2d, 4th, and 9th of May, Dr. Park lectured on the Theory and Phenomena of Fever. On the 11th, 16th, and 18th of May, Dr. Cooke on Epilepsy. On the 23d, 25th, and 30th of May, Dr. Powel delivered the Lumleian Lectures; and on the 1st, 6th, 8th, and 13th of June, Dr. Paris will give Lectures on the Collection of Materia Medica.

At the College of Surgeons Messrs. Wilson and Brodie are lecturing—the former on the Urinary and Genital Organs—the latter on Comparative Anatomy.

A second edition (Italian) of Professor Scarpa's Work on Hernia has lately been published, enlarged by the introduction of many important anatomical and pathological observations, as well as practical rules in surgery, drawn from the experience of himself and others. It is printed distinct from the plates, and at a low price.

Dr. Forbes, of Penzance, has nearly ready for publication a *condensed* translation of Lannæc's work on Diseases of the Chest, which will be enriched with notes and illustrations by the translator.

### THEATRE OF ANATOMY, MEDICINE, &c.

*Blenheim Street, Great Marlborough Street.*

The Summer Course of Lectures, at this School, will begin on the following days:—

Anatomy, Physiology, and Surgery, by Joshua Brookes, F.R.S. daily, at Seven in the Morning, on Friday, June 6th.—Dissections as usual.

Chemistry, Materia Medica, &c. daily, at Eight in the Morning; Theory and Practice of Physic, at Nine, with Examinations, by Dr. Ager, on Monday, June 4th.

Three Courses are given every year, each occupying nearly four months.—Further particulars may be known of Mr. Brookes, at the Theatre; or of Dr. Ager, 69, Margaret-street, Cavendish-square.

---

### MEDICAL PUPIL.

Dr. James Johnson will admit into his family, on the 1st of October next, for one or more years, a young gentleman pursuing his professional studies in London. The terms, which are moderate, may be known by application to Dr. J.

☞ *M. is requested to transmit the documents.*

*Additional Subscribers since last Quarter.*

Anderson, Mr. Surgeon, R. N.
Babington, Dr. Aldermanbury
Baltimore Medical Society, United States
Bateman, Geo. Esq. Surgeon, Yarmouth
Bowden, Stephen, Esq. Surgeon, R. N. Plymouth
Cross, Mr. John, Surg. Norwich
Chester Medical Society
Cooper, Dr. Dorchester
Caldwell, Charles, M. D. Dean of Transylvania University, Lexington, Kentucky
Cample, John, M. D. Surgeon, Ashton-under-Line
Clarke, Mr. Surgeon, Lincoln's-Inn-fields
Daubeny, C. (M. D.) Oxford
Davids, Mr. Surg. West Cowes, Isle of Wight
Deverill, G. S. Esq. at Dr. Ballingall's, Howe Street, Edinb.
Dawson, Messrs. Surg. Wakefield, Yorkshire
Dennett, John Thomas, L. S. A. Surgeon, Bognor
Evans, Mr. Surgeon, Belper, Derbyshire
Flemming, Dr. John, M. P. Gloucester-place
Fergusson, Mr. R. N.
Furber, G. Esq. Memb. Royal Coll. Surg. Malpas, Cheshire
Fraser, Dr. Doughty Street
Freeman, Mr. Surg. Bracknell, Berks
Gannon, Mr. John, Assist. Surg. H. M. S. Medina
Grigg, Mr. Surgeon, R. N.
Griffiths, Dr. Royal Navy
Gibney, Dr. Wm. Cheltenham
Godfrey, Mr. Surg. Chenlbury, Oxfordshire
Granger, B. Esq. Surgeon, Burton-on-Trent
George, Henry, Esq. Surgeon, Kensington.
Godwin, Mr.
Hutchisson, Dr. Guernsey
Hunter, Dr. Adam, Leeds
Herberski, Profes. Vincent, University of Wilna, Lithuania
Hammond, Mr. C. C. Surgeon, Ipswich
Irvine, Mr. S. Surgeon, H.M.S. Sophy, to East Indies
Jackson, Mr. (place not stated)
James, Dr. D. Bourdeaux
Johnson, Mr. at London Hospital
Keys, Thomas, Esq. Assistant-Surgeon, Madras
Knight, Mr. Paulslade, Governor of Lancaster County Asylum
Kenny, Mr. (Student)
Loudon, J. Esq. Surg. H. M. S. Doris
Leeds Medical Society
Lexington Library, Kentucky, America
Martin, Dr. Peter, Dunning, Perthshire
M'Lean, Mr. Jos. Surg. H.M.S. Medina, Halifax
Miller, Dr. E. Londonderry
Morris, Mr. Thomas, Surgeon, Ashton-under-Line
Northen, Dr. Newcastle-under-Line
Palin, Ralph, M. D. Newport, Shropshire
Ruxton, John, Esq. Ensham Hall, Oxen
Swain, Jas. Esq. Upper Charles Street, Northampton Square
Sexty, Mr. Surgeon, West Kent Militia, Maidstone
Shillito, Mr. Charles, Surgeon, Putney
Shand, Dr. Royal Navy, C. G. Hope
Semple, Mr. Surgeon
Tolmie, James, Esq. Surg. Cambletown, Fort George, N. B.
Thomson, Henry Benwell, Esq. of the Royal College of Surgeons, and one of the Surgeons to the Kent Dispensary
Thomas, Dr. Cheltenham
Taylor, W. Esq. Surg. North Yarmouth
Turner, Mr. Surgeon, Stanley Bridge, Ashton-under-Line
Watling, Thomas, Esq. Member of the Royal College of Surgeons, Leominster

